# Effectiveness of cilostazol in transient ischemic attack refractory to aspirin: A report of two cases

**DOI:** 10.3892/etm.2013.1468

**Published:** 2013-12-31

**Authors:** GAOPING LIN, DONGDONG REN, SHUNYUAN GUO, YU GENG

**Affiliations:** Department of Neurology, Zhejiang Provincial People’s Hospital, Hangzhou, Zhejiang 310014, P.R. China

**Keywords:** transient ischemic attack, cilostazol, aspirin

## Abstract

Transient ischemic attack (TIA) is a warning of impending ischemic stroke. It provides an important therapeutic time window in which appropriate intervention may prevent permanent neurological injury. The anti-platelet agent, aspirin, is an option for reducing the risk of stroke following TIA. However, for patients who are not responsive to aspirin, cilostazol may be an effective treatment. The current study presents two cases of TIA that were refractory to aspirin but were successfully treated with cilostazol. In case 1, an 83-year-old female patient suffered from episodes of weakness and numbness of the left extremities. Aspirin alone or aspirin in combination with clopidogrel were not effective. Anticoagulation therapy with low molecular heparin decreased the frequency of ischemic episodes with complete remission following antiplatelet therapy with cilostazol. In case 2, a 51-year-old male presentedwith episodes of paroxysmal weakness of the left extremities with dysarthria. Antiplatelet therapy with aspirin was initiated. Eight episodes of ischemic attack recurred on the seventh day following admission. After the change of the antiplatelet agent to cilostazol, no ischemic episodes recurred, with the exception of three on the first day. This study suggested that cilostazol may be efficacious in the prevention of ischemic stoke following TIA of a non-cardiac origin that was not responsive to aspirin.

## Introduction

Transient ischemic attack (TIA) is an important risk factor for ischemic stroke. Appropriate antithrombotic therapy may reduce the risk of stroke following TIA. Aspirin is the antiplatelet agent most widely used to treat TIA of noncardioembolic origin and it is able to reduce the risk of stroke by 19% in patients who have experienced TIA or a stroke (1). However, aspirin is not able to thoroughly control TIA in certain patients. Another antiplatelet agent, cilostazol, may be considered as an alternative treatment in these cases. Cilostazol is a quinolinone-derivative medication widely used in patients with peripheral vascular disease. It is a phosphodiesterase inhibitor which inhibits platelet aggregation. This study reports two cases of TIA refractory to aspirin and the effectiveness of cilostazol in these cases.

## Case reports

### Case 1

**Figure 1:**
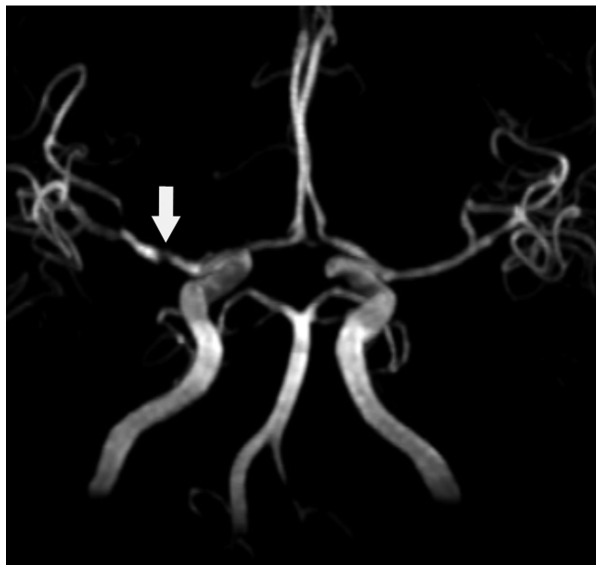
Case 1. Magnetic resonance angiography showed severe segmental stenosis of the right middle cerebral artery.

An 83-year-old female patient was admitted to the Zhejiang Provincial People’s Hospital (Hangzhou, China) due to episodes of weakness and numbness of the left extremities during the previous 20 days. These episodes lasted ~10 min and occurred 8–10 times every day. Consciousness was never impaired and the patient experienced no dizziness or additional complaints. The patient had a past history of diabetes and hypertension, which were treated with glimepiride and felodipine, respectively. Physical examination showed no neurological deficits. Magnetic resonance angiography revealed severe segmental stenosis of the right middle cerebral artery (Fig. 1). No new infarcts were observed following diffusion-weighted magnetic resonance imaging. Sinus rhythm was observed in the electrocardiogram and mild aortic valve regurgitation with no thrombus was reflected in the echocardiogram. Carotid ultrasound showed no atherosclerotic plaques. The low density lipoprotein cholesterol level was 2.91 mmol/l. A diagnosis of TIA was made.

The patient was treated with 200 mg aspirin and 20 mg atorvastatin once per day for 7 days. Episodes of weakness and numbness of the left extremities continued to occur 7–10 times per day. Clopidogrel (75 mg) was then administered once per day; however, episode frequency did not decrease. On the 11th day following admission, anticoagulation therapy with low molecular heparin (4100 U) twice a day replaced antiplatelet therapy. The frequency of episodes decreased to once or twice a day. On the 19th day following admission, 100 mg cilostazol was administered twice a day in place of the anticoagulation therapy. No symptoms of TIA were observed from then on during the three-year follow-up period, with the exception of one episode of perioral numbness of the left side lasting for 1 min on the first day following the adjustment of medication.

### Case 2

A 51-year-old male presented to the Zhejiang Provincial People’s Hospital (Hangzhou, China) due to two episodes of paroxysmal weakness of the left extremities with dysarthria during the previous 30 h. The first episode lasted for 10 min and the second one for 3 min. The patient had a history of hypertension and was treated with losartan, and a history of diabetes mellitus, which was controlled successfully with gliclazide. No neurological deficits were found on physical examination. Diffusion-weighted magnetic resonance imaging showed no signs of acute infarction. Computed tomography perfusion of the brain was normal. Ultrasonography revealed atherosclerosis of both carotid arteries. Electrocardiogram showed sinus rhythm and no thrombus was found following the echocardiogram. The low density lipoprotein cholesterol level was 3.39 mmol/l. Digital subtraction angiography reflected mild stenosis of the right internal carotid artery. The patient was diagnosed with TIA.

Antiplatelet therapy with aspirin and anti-atherosclerotic therapy with atorvastatin were initiated. On the seventh day following admission, episodes of weakness of the left extremities with dysarthria recurred. These episodes lasted for 2–3 min each time and occurred eight times in total on that day. The antiplatelet agent was replaced with 100 mg cilostazol twice a day. On the first day after treatment adjustment, three episodes of ischemia similar to the previous ones recurred. Diffusion-weighted magnetic resonance imaging excluded new cerebral infarction. No weakness of the limbs or dysarthria recurred from then on during the two-year follow-up period.

## Discussion

With the development of neuroimaging techniques, the time criterion for TIA has been challenged. In one study, it was identified that approximately one-third of patients that were clinically diagnosed with TIA showed associated new infarcts following MRI (2). A formal evidence review suggested a new definition of TIA as ‘a brief episode of neurological dysfunction caused by focal brain, spinal cord or retinal ischemia, without acute infarction’ (3). It focuses on whether histological damage occurs. Diffusion-weighted magnetic resonance imaging of the two patients in the present study was normal, which supported the diagnosis of TIA.

The early risk of ischemic stroke following TIA is very high. The stroke risk following TIA has been estimated to be 8.0% within seven days, 11.5% within one month and 17.3% within three months (4). Several scoring systems have been designed to evaluate the risk of infarction following TIA. The ABCD2 score is a popular and effective predictor, which is based on age, blood pressure, clinical features, the duration of symptoms and diabetes mellitus. An ABCD2 score of ≥4 is an independent predictor of the risk of stroke one week and 90 days following the onset of TIA (5). The ABCD2 scores of the case 1 and 2 patients were 6 and 5, respectively. Both patients were at high risk of infarction.

A previous study has shown that urgent evaluation and treatment may lower the risk of stroke in 90 days, subsequent hospital bed-days, acute costs and 6-month disability (6). However, the majority of randomized controlled trials on the secondary prevention of ischemic stroke have included patients with TIA and infarction rather than patients with TIA only (7,8). Therefore, the best antithrombotic regimen for TIA remains unclear. The 9th edition of the guidelines for antithrombotic therapy and prevention of thrombosis issued by the American College of Chest Physicians recommended long-term treatment with aspirin, clopidogrel, aspirin/extended-release dipyridamole or cilostazol in patients with a history of noncardioembolic ischemic stroke or TIA (9). The Chinese Acute Stroke Trial (CAST) (10) and the International Stroke Trial (IST) (11) demonstrated that initiating antiplatelet therapy with aspirin soon after the onset of acute ischemic stroke reduced the risk of recurrent stroke or death. The Fast assessment of stroke and TIA to prevent early recurrence (FASTER) trial (12) implied that a combination of aspirin and clopidogrel may be more effective than aspirin alone following TIA or a minor stroke within 90 days.

The effects of anticoagulation therapy for non-cardiac TIA have not been fully evaluated. In the IST (11), heparin significantly decreased recurrent ischemic stroke following cerebral infarction, but increased hemorrhage with no net benefit. Therefore, heparin is not recommended for routine use following ischemic stroke. It should only be considered in patients at high risk of early recurrence.

The patient of case 1 had TIA of non-cardiac origin. Aspirin alone or aspirin in combination with clopidogrel were not effective. Anticoagulation therapy with low molecular heparin decreased the frequency of ischemic episodes with complete remission following antiplatelet therapy with cilostazol. In case 2, eight episodes of weakness of the left extremities with dysarthria recurred while the patient was treated with aspirin. Following the change of the antiplatelet agent to cilostazol, no ischemic episodes recurred, with the exception of three on the first day.

Cilostazol is a phosphodiesterase type 3 inhibitor, which increases the cyclic adenosine monophosphate concentration inside platelets, which subsequently potentiates the inhibitory signals of glycoprotein, IIb–IIIa. No randomized controlled trials evaluating the role of cilostazol in the prevention of ischemic stroke following TIA have been published. However, the effects of cilostazol as a secondary prevention after ischemic stroke have been evaluated. Compared with aspirin, cilostazol is equal to or more effective in the secondary prevention of stroke after noncardioembolic stroke (13). It is particularly effective for lacunar infarction and ischemic stroke with hypertension or diabetes mellitus (14). In a pilot study, intracranial hemorrhage was observed to be less common in patients treated with cilostazol than in those treated with aspirin (15). However, cilostazol caused a greater incidence of side-effects, including headache, diarrhea, palpitations, dizziness and tachycardia. An escalation regimen of cilostazol starting from 50 mg twice a day for an initial 4 days followed by 100 mg twice a day may increase tolerability (16).

Cilostazol was particularly efficacious for the two patients with TIA in the present study. Cilostazol not only inhibits platelet aggregation, but also has an effect of vasodilation by increasing the production of nitric oxide and reducing intracellular calcium ion concentrations (17). Moreover, it protects the endothelium and inhibits smooth muscle proliferation of the vascular walls (18).

In conclusion, cilostazol was particularly effective for the two patients with TIA in the present study. It may be efficacious in the prevention of ischemic stoke following TIA of a non-cardiac origin that is not responsive to aspirin.
